# Associations between systemic immune-inflammation index and immune-related hypothyroidism in non-small cell lung cancer patients receiving immune checkpoint inhibitors: a retrospective study

**DOI:** 10.3389/fonc.2026.1844002

**Published:** 2026-06-02

**Authors:** Wei Dai, Qian Li, Meijuan Wang, Chunbin Wang, Honggang Cao

**Affiliations:** 1 Department of Oncology, Affiliated Hospital 6 of Nantong University, Yancheng Third People’s Hospital, Yancheng, Jiangsu, China; 2 Department of Rehabilitation Medicine, Affiliated Hospital 6 of Nantong University, Yancheng Third People’s Hospital, Yancheng Jiangsu, China

**Keywords:** immune checkpoint inhibitors, immune-related hypothyroidism, non-small cell lung cancer, predictive model, systemic immune-inflammation index

## Abstract

**Objective:**

Immune-related hypothyroidism (irH) is one of the most common endocrine immune-related adverse events (irAEs) mediated by immune checkpoint inhibitors (ICIs), and its occurrence is influenced by the systemic immune-inflammatory status. This study investigated the association between baseline systemic immune-inflammation index (SII) and irH risk in patients with non-small cell lung cancer (NSCLC) receiving ICIs.

**Methods:**

In this retrospective study, patients with driver gene-negative locally advanced or metastatic NSCLC were included. All patients underwent first-line treatment with PD-1 inhibitors combined with chemotherapy at Yancheng Third People’s Hospital between October 2019 and December 2024. SII was determined using the following formula: (platelet count × neutrophil count)/lymphocyte count. Univariate and multivariate logistic regression analyses were performed to determine independent factors associated with irH. A nomogram prediction model was subsequently constructed and assessed using calibration curves, receiver operating characteristic (ROC) curves, and decision curve analysis (DCA).

**Results:**

160 patients were enrolled, including 65 (40.6%) in the irH group and 95 (59.4%) in the non-irH group. Compared with the non-irH group, the irH group had significantly lower baseline log2-SII (8.95 ± 0.65 vs. 9.55 ± 0.80, P < 0.001). Multivariate regression analysis revealed that log2-SII (OR = 0.13, 95% CI: 0.05-0.27, P < 0.001) was an independent protective predictor for irH, while age, TG, LDL, TB, FIB, and D-dimer were independent predictors (all P < 0.05). The nomogram developed from these factors demonstrated potential discriminative ability (AUC = 0.884, 95% CI: 0.832–0.935) and calibration (absolute error = 0.025), and DCA revealed a net clinical benefit across a threshold probability range of 5%–90%. Subgroup analysis revealed that in both LUSC and LUAD patients, the irH group exhibited significantly lower log2-SII levels than the non-irH group (all P < 0.05).

**Conclusion:**

SII is closely associated with irH in NSCLC patients receiving ICIs, with baseline SII serving as an independent predictor of irH. The nomogram incorporating SII along with age, lipid profiles, bilirubin, and coagulation parameters can effectively predict the risk of irH, providing a reference for identifying high-risk patients at an early stage and enhanced thyroid function monitoring in clinical practice.

## 1 Introduction

Lung cancer remains the most commonly diagnosed malignancy and the leading cause of cancer-related death worldwide and in China, posing a significant threat to public health (1, 2). Non-small cell lung cancer (NSCLC) represents the most prevalent pathological type, comprising approximately 85% of all lung cancer cases. In China, about 75% of NSCLC patients have lost the opportunity for curative surgery or even present with distant metastasis at initial diagnosis, resulting in poor long-term survival outcomes and an overall unfavorable prognosis in advanced-stage disease (3). Currently, the combination of programmed cell death protein 1 (PD-1) and its ligand (programmed death-ligand 1, PD-L1) inhibitors with conventional chemotherapy has become one of the standard first-line treatment options for patients with driver gene-negative locally advanced (inoperable and unsuitable for radiotherapy) and advanced metastatic NSCLC (4). However, while ICIs activate T-cell-mediated antitumor immune responses, they can also lead to the immune system attacking normal tissues and organs, thereby triggering a range of immune-related adverse events (irAEs). Among these, immune-related thyroid dysfunction (irTD), as the most representative immune-related endocrine adverse reaction, is particularly common in clinical practice, predominantly presenting as immune-related hypothyroidism (irH) (5). The incidence of irH ranges between 6% and 20% (6). Common symptoms in affected patients include fatigue, alopecia, and depressed mood, which may significantly impair treatment adherence and quality of life.

Studies have confirmed that factors such as sex, baseline thyroid peroxidase antibodies (TPO-Ab), thyroglobulin antibodies (TgAb), and baseline thyroid-stimulating hormone (TSH) are closely associated with the occurrence of irTD (7, 8). This suggests that the occurrence of irH is not an isolated adverse drug reaction event; rather, it may involve more complex mechanisms of host immune response and inflammatory regulation. The antitumor effects of ICIs and the thyroid damage they induce are, in essence, two distinct manifestations of the widespread activation of the immune system, both of which are closely associated with the immune-inflammatory status in the systemic circulation and in the tumor microenvironment (9, 10). Therefore, identifying biomarkers that can effectively reflect the systemic immune-inflammatory burden is of great significance for understanding the pathogenesis of irH and its association with therapeutic efficacy.

The systemic immune-inflammation index (SII) is a novel inflammatory marker that integrates peripheral blood platelet, neutrophil, and lymphocyte counts. Compared with single parameters, SII offers a more comprehensive evaluation of the body’s inflammatory and immune status (11). Given that the occurrence of irH is strongly associated with immune activation, and SII serves as a sensitive indicator of this immune-inflammatory state, we examined the association between SII and irH in NSCLC patients receiving immunotherapy-containing regimens. This investigation may contribute to improved clinical risk prediction and the management of irH.

## 2 Methods

### 2.1 Study population

**Figure 1:**
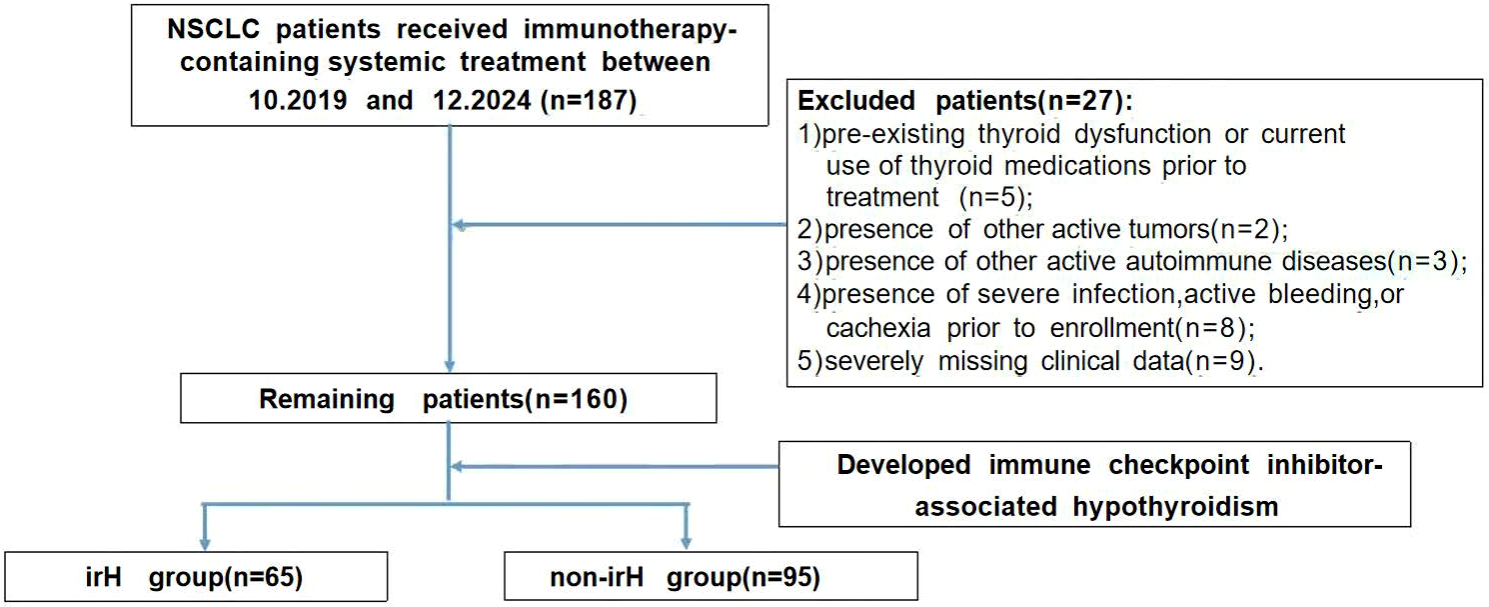
Research flowchart of the study.

This retrospective analysis comprised 187 patients with locally advanced or advanced NSCLC who received immunotherapy-containing systemic treatment at Yancheng Third People’s Hospital between October 1, 2019 and December 31, 2024. Patients were categorized into two groups according to the occurrence of irH during treatment: the irH group and the non-irH group. The irH group only included patients who developed hypothyroidism as an irAE, and no patients in this group experienced immune−related adverse reactions such as dermatitis or hepatitis. In additional, no patients in the non-irH group developed immune-related adverse reactions including dermatitis or hepatitis. Studies were included if they met the following criteria: (1) patients were histologically/cytologically and radiologically confirmed as having locally unresectable or advanced NSCLC; (2) negative for driver gene alterations including EGFR, ALK, ROS-1, RET, MET, NTRK, and BRAF V600; (3) received first-line treatment with PD-1 inhibitors combined with conventional chemotherapy; (4) completed a minimum of two cycles of standard treatment; (5) had an Eastern Cooperative Oncology Group (ECOG) performance status score of 0-1. The following criteria were applied for exclusion: (1) pre-existing thyroid dysfunction or current use of thyroid medications prior to treatment; (2) presence of other active tumors; (3) presence of other active autoimmune diseases; (4) presence of severe infection, active bleeding, or cachexia prior to enrollment; (5) missing key clinical data. **Figure 1** illustrates the patient selection process. In strict accordance with the exclusion criteria, we excluded with pre-existing thyroid dysfunction or those using thyroid medications before treatment (n=5), other active malignancies (n=2), other active autoimmune diseases (n=3) and severe infection, active bleeding, or cachexia before enrollment (n=8). In addition, to ensure the reliability of the results, we removed all unclear or missing data (n=9). A total of 27 patients were excluded. The study complied with the Declaration of Helsinki and was approved by the Ethics Committee of Yancheng Third People’s Hospital (Approval No. 2025-87).

### 2.2 Data collection

Patient general characteristics (age, sex, weight, height, BMI), lifestyle-related factors (smoking and drinking history), underlying diseases (history of hypertension, diabetes, coronary heart disease), clinical data (pathological type), and laboratory parameters were obtained from the electronic medical record system of Yancheng Third People’s Hospital. Fasting venous blood samples (3 mL) were collected and placed into appropriate tubes as follows: (1) Peripheral blood cell counts were measured using an automatic hematology analyzer (XN-10(B2), Sysmex, Japan) with EDTA-anticoagulated whole blood. (2) Coagulation parameters were analyzed using an automatic coagulation analyzer (CS-5100, Sysmex, Japan) on plasma separated from sodium citrate-anticoagulated blood by centrifugation at 3,000 rpm for 15 minutes. (3) Biochemical and lipid profiles were determined using an automatic biochemical analyzer (cobas c701/c702, Roche, Germany) on serum separated from blood collected in plain tubes with clot activator by centrifugation at 3,000 rpm for 15 minutes. All blood samples were obtained at baseline before the start of immunotherapy combined with chemotherapy, with patients under fasting conditions.

### 2.3 Treatment: PD-1 inhibitor + chemotherapy

In this study, all patients received immunotherapy-containing combination treatment, and no patients received immunotherapy alone. The PD-1 inhibitors used in this study included camrelizumab, tislelizumab, sintilimab, and toripalimab. Chemotherapy regimens were determined according to pathological type. For lung squamous cell carcinoma (LUSC), chemotherapy consisted of taxanes, gemcitabine, or vinorelbine, with or without platinum-based agents. For lung adenocarcinoma (LUAD), chemotherapy consisted of pemetrexed or taxanes, with or without platinum-based agents. Taxanes specifically included docetaxel, paclitaxel, nanoparticle albumin-bound paclitaxel, and liposomal paclitaxel. Platinum-based agents included cisplatin, carboplatin, nedaplatin, and lobaplatin.

### 2.4 Outcome variable: irH

In this study, irH specifically refers to primary hypothyroidism. According to the diagnostic standards for primary hypothyroidism and the literature of immune-related endocrine toxic effects, the laboratory diagnosis criteria of irH in this study were as follows: During treatment with PD-1 inhibitor-containing regimens and within three months after cessation, patients experienced one of the following situations: (1) Primary hypothyroidism: elevated serum thyroid-stimulating hormone (TSH) levels combined with reduced serum free thyroxine (FT4) levels; (2) Subclinical hypothyroidism: elevated serum TSH levels (under 10 mU/L), while FT4 levels were within the normal range (12, 13).

### 2.5 Exposure variable: SII

SII, as a valuable indicator reflecting systemic inflammation and immunological status, was expressed using the following equation: SII = (platelet count × neutrophil count)/lymphocyte count (11, 14). Due to the uneven distribution of SII, its value was log-2 transformed for subsequent analysis. The choice of log2 specifically was supported by multiple recent studies that have employed log2-transformed SII in similar biomedical analyses (14–16).

### 2.6 Management of irH

All patients who developed irH were managed according to a standardized institutional protocol. Thyroid function tests, including serum thyroid−stimulating hormone (TSH) and free thyroxine (FT3, FT4), were monitored at baseline prior to each cycle of immunotherapy and whenever clinical symptoms suggestive of thyroid dysfunction were present. For irH patients, those with mild TSH elevation (TSH 5-10 mU/L) who remained asymptomatic were closely observed with repeated thyroid function monitoring. Symptomatic hypothyroid patients with TSH > 10 mU/L received levothyroxine replacement therapy, and the dose was adjusted to maintain euthyroidism. Levothyroxine was initiated at a dose of 25–50 μg/day, individualized based on patient age, body weight, and baseline TSH level. Dose adjustments were made every 4 weeks according to follow-up thyroid function tests until TSH levels stabilized within the normal range. Furthermore, because thyroid-related irAEs rarely require high-dose systemic corticosteroid therapy, and previous studies have shown that early use of high-dose corticosteroids after irAE onset is associated with significantly worse overall survival (17, 18), no corticosteroids were administered in this cohort. In addition, because glucocorticoids can affect SII values, we further confirm that no patient in this study received systemic corticosteroids for brain metastases or any other immune-related adverse reactions. Patients with lung cancer brain metastases requiring symptomatic glucocorticoid therapy (e.g., for mass effect, brain edema, or elevated intracranial pressure) were not included. Moreover, no patient developed other irAEs (e.g., dermatitis, hepatitis, colitis, pneumonitis) that would necessitate corticosteroid use. Therefore, steroid-related confounding of SII is unlikely in this cohort. All management decisions were made by a multidisciplinary team, including oncologists and endocrinologists.

### 2.7 Sample size calculation

This study employed logistic regression analysis. For logistic regression, it is generally required that each outcome category contain at least 50 samples, and bias in parameter estimation becomes negligible when the total sample size exceeds 200. According to the common rule of thumb, the total sample size should exceed 100 cases and be 5–10 times the number of predictor variables. In the present study, the final model included seven predictor variables; therefore, a minimum of 100 cases was required (19). The total sample size of this study was 160 cases, which conforms to this rule of thumb.

### 2.8 Statistical analysis

Excel 2025 was used to record the data, which were then analysed with R software (4.4.3). The R packages utilized included “compareGroups”, “forestmodel”, “rms”, “pROC”, and “rmda”. Frequencies and percentages (n, %) were used to summarize categorical variables, while continuous variables were reported as mean ± standard deviation or as median with interquartile range (P25, P75). Patients were categorized into irH and non-irH groups according to the occurrence of irH. Continuous variables were compared between groups using the t-test or Mann–Whitney U test, while categorical variables were assessed using the chi-square test or Fisher’s exact test. Variables with P < 0.05 in univariate logistic regression were entered into multivariate logistic regression analysis. Independent predictors were identified using a backward stepwise selection approach and then used to construct a nomogram. The predictive performance of the model was examined by ROC curves, calibration curves, and decision curve analysis (DCA). All analyses were conducted using two-sided tests, with P < 0.05 indicating statistical significance.

## 3 Results

### 3.1 Baseline characteristics

Ultimately, the study population consisted of 160 patients, of whom 133 were male (83.12%) and 27 were female (16.88%). The mean age was 67.06 ± 8.01 years, and the mean BMI was 22.95 ± 3.62 kg/m². A total of 64 (40.00%) reported a history of smoking, and 34 (21.25%) reported a history of drinking. Regarding pathological types, 97 patients (60.62%) had lung squamous cell carcinoma (LUSC) and 63 patients (39.38%) had lung adenocarcinoma (LUAD).

The irH group showed significantly elevated levels of age, LDL, TG, TC, FBG, PT, FIB, and D-dimer compared with the non-irH group (all P < 0.05). Conversely, log2-SII was reduced in the irH group (8.95 ± 0.65 vs 9.55 ± 0.80, P < 0.001). Details were presented in **Table 1**.

**Table 1:** Overall patient baseline.

Variable	Total (n=160)	Non-irH group (n=95)	irH group (n=65)	P
Gender, n (%)				0.129
Male	133 (83.12%)	83 (87.37%)	50 (76.92%)	
Female	27 (16.88%)	12 (12.63%)	15 (23.08%)	
Age (years)	67.06 ± 8.01	65.96 ± 7.89	68.68 ± 7.96	0.035
Height (cm)	166.62 ± 6.96	166.76 ± 6.80	166.43 ± 7.24	0.774
Weight (kg)	63.65 ± 10.20	63.15 ± 10.50	64.38 ± 9.77	0.451
BMI (kg/m^2)	22.95 ± 3.62	22.72 ± 3.62	23.30 ± 3.62	0.322
Smoking History, n (%)				0.139
No	96 (60.00%)	62 (65.26%)	34 (52.31%)	
Yes	64 (40.00%)	33 (34.74%)	31 (47.69%)	
Drinking History, n (%)				0.065
No	126 (78.75%)	80 (84.21%)	46 (70.77%)	
Yes	34 (21.25%)	15 (15.79%)	19 (29.23%)	
Hypertension History, n (%)				0.115
No	99 (61.88%)	64 (67.37%)	35 (53.85%)	
Yes	61 (38.12%)	31 (32.63%)	30 (46.15%)	
Diabetes History, n (%)				0.375
No	141 (88.12%)	86 (90.53%)	55 (84.62%)	
Yes	19 (11.88%)	9 (9.47%)	10 (15.38%)	
Coronary Heart Disease History, n (%)				0.567
No	157 (98.12%)	94 (98.95%)	63 (96.92%)	
Yes	3 (1.88%)	1 (1.05%)	2 (3.08%)	
Pathological Type, n (%)				0.490
LUSC	97 (60.62%)	55 (57.89%)	42 (64.62%)	
LUAD	63 (39.38%)	40 (42.11%)	23 (35.38%)	
Laboratory results				
WBC (10^9/L)	6.54 ± 1.72	6.41 ± 1.76	6.73 ± 1.64	0.239
RBC (10^12/L)	4.08 ± 0.62	4.11 ± 0.58	4.04 ± 0.69	0.514
MCV (fL)	91.99 ± 5.20	91.84 ± 5.01	92.22 ± 5.50	0.655
MCHC (g/L)	324.52 ± 11.34	325.32 ± 11.23	323.35 ± 11.48	0.283
Hemoglobin (g/L)	121.29 ± 19.35	122.28 ± 18.26	119.85 ± 20.89	0.447
Neutrophil count (10^9/L)	4.37 ± 1.43	4.58 ± 1.54	4.05 ± 1.20	0.014
Lymphocyte count (10^9/L)	1.22 ± 0.44	1.14 ± 0.37	1.33 ± 0.51	0.013
Monocyte count (10^9/L)	0.53 ± 0.27	0.54 ± 0.29	0.52 ± 0.23	0.673
Platelet count (10^9/L)	187.40 ± 66.57	199.19 ± 70.27	170.17 ± 57.01	0.005
SII	743.73 ± 478.75	880.61 ± 550.20	543.67 ± 237.37	<0.001
log2-SII	9.31 ± 0.80	9.55 ± 0.80	8.95 ± 0.65	<0.001
HDL (mmol/L)	1.16 ± 0.27	1.19 ± 0.27	1.13 ± 0.25	0.167
LDL (mmol/L)	2.38 ± 0.73	2.25 ± 0.69	2.57 ± 0.75	0.008
TG (mmol/L)	1.46 ± 0.61	1.35 ± 0.53	1.61 ± 0.69	0.010
TC (mmol/L)	4.16 ± 0.81	4.03 ± 0.78	4.35 ± 0.82	0.016
FBG (mmol/L)	5.54 ± 1.50	5.29 ± 1.03	5.90 ± 1.95	0.023
ALT (U/L)	20.83 ± 13.32	20.89 ± 12.60	20.75 ± 14.41	0.950
AST (U/L)	22.42 ± 8.75	22.48 ± 8.15	22.33 ± 9.63	0.918
TB (umol/L)	9.64 ± 3.49	9.17 ± 3.02	10.32 ± 4.01	0.051
TP (g/L)	67.55 ± 5.73	67.49 ± 5.55	67.65 ± 6.04	0.860
PT (s)	11.56 ± 0.97	11.43 ± 0.84	11.75 ± 1.10	0.049
APTT (s)	27.25 ± 2.21	27.25 ± 1.85	27.26 ± 2.67	0.976
FIB (g/L)	4.25 ± 1.31	4.03 ± 1.36	4.56 ± 1.18	0.010
D-dimer (mg/L FEU)	1.12 ± 0.74	0.94 ± 0.54	1.38 ± 0.90	0.001

Continuous variables were expressed as Mean ± SD and categorical variables were expressed as NO. (%). irH, immune-related hypothyroidism; BMI, body mass index; LUSC, lung squamous cell carcinoma; LUAD, lung adenocarcinoma; WBC, white blood cell; RBC, red blood cell; MCV, mean corpuscular volume; MCHC, mean corpuscular hemoglobin concentration; SII, systemic immune-inflammation index; HDL, high density lipoprotein; LDL, low density lipoprotein; TG, triglycerides; TC, total cholesterol; FBG, fasting blood glucose; ALT, alanine aminotransferase; AST, aspartate aminotransferase; TB, total bilirubin; TP, total protein; PT, prothrombin time; APTT, activated partial thromboplastin time; FIB, fibrinogen.

### 3.2 Univariate logistic regression analysis

As shown in **Table 2**, univariate logistic regression analysis highlighted several variables potentially linked to the risk of irH. These included age (OR: 1.05, 95% CI: 1.00-1.09, P = 0.038), drinking (OR: 2.19, 95% CI: 1.01-4.80, P = 0.046), log2-SII (OR: 0.32, 95% CI: 0.19-0.53, P < 0.001), LDL (OR: 1.89, 95% CI: 1.17-3.05, P = 0.009), TG (OR: 2.12, 95% CI: 1.18-3.79, P = 0.012), TC (OR: 1.66, 95% CI: 1.09-2.53, P = 0.019), FBG (OR: 1.37, 95% CI: 1.05-1.78, P = 0.020), TB (OR: 1.10, 95% CI: 1.00-1.21, P = 0.042), PT (OR: 1.42, 95% CI: 1.01-1.99, P = 0.041), FIB (OR: 1.38, 95% CI: 1.07-1.77, P = 0.013), and D-dimer (OR: 2.46, 95% CI: 1.45-4.18, P = 0.001).

**Table 2:** Univariate logistic regression analysis.

Variable	OR (95% CI)	P
Gender, n (%)		
Male	Reference	
Female	2.06 (0.89, 4.88)	0.092
Age (years)	1.05 (1.00, 1.09)	0.038
BMI (kg/m^2)	1.05 (0.96, 1.14)	0.321
Smoking History, n (%)		
No	Reference	
Yes	1.71 (0.89, 3.27)	0.105
Drinking History, n (%)		
No	Reference	
Yes	2.19 (1.01, 4.80)	0.046
Hypertension History, n (%)		
No	Reference	
Yes	1.76 (0.92, 3.40)	0.088
Diabetes Mellitus History, n (%)		
No	Reference	
Yes	1.73 (0.65, 4.67)	0.271
Coronary Heart Disease History, n (%)		
No	Reference	
Yes	2.79 (0.22, 88.9)	0.425
Pathological Type, n (%)		
LUSC	Reference	
LUAD	0.76 (0.39, 1.45)	0.400
WBC (10^9/L)	1.12 (0.93, 1.34)	0.245
RBC (10^12/L)	0.84 (0.50, 1.40)	0.497
MCV (fL)	1.01 (0.95, 1.08)	0.647
MCHC (g/L)	0.98 (0.96, 1.01)	0.280
Hemoglobin (g/L)	0.99 (0.98, 1.01)	0.433
log2-SII	0.32 (0.19, 0.53)	<0.001
HDL (mmol/L)	0.43 (0.13, 1.46)	0.175
LDL (mmol/L)	1.89 (1.17, 3.05)	0.009
TG (mmol/L)	2.12 (1.18, 3.79)	0.012
TC (mmol/L)	1.66 (1.09, 2.53)	0.019
FBG (mmol/L)	1.37 (1.05, 1.78)	0.020
ALT (U/L)	1.00 (0.98, 1.02)	0.948
AST (U/L)	1.00 (0.96, 1.03)	0.915
TB (umol/L)	1.10 (1.00, 1.21)	0.042
TP (g/L)	1.01 (0.95, 1.06)	0.857
PT (s)	1.42 (1.01, 1.99)	0.041
APTT (s)	1.00 (0.87, 1.16)	0.974
FIB (g/L)	1.38 (1.07, 1.77)	0.013
D-dimer (mg/L FEU)	2.46 (1.45, 4.18)	0.001

BMI, body mass index; LUSC, lung squamous cell carcinoma; LUAD, lung adenocarcinoma; WBC, white blood cell; RBC, red blood cell; MCV, mean corpuscular volume; MCHC, mean corpuscular hemoglobin concentration; SII, systemic immune-inflammation index; HDL, high density lipoprotein; LDL, low density lipoprotein; TG, triglycerides; TC, total cholesterol; FBG, fasting blood glucose; ALT, alanine aminotransferase; AST, aspartate aminotransferase; TB, total bilirubin; TP, total protein; PT, prothrombin time; APTT, activated partial thromboplastin time; FIB, fibrinogen.

### 3.3 Multiple logistic regression analysis

**Figure 2:**
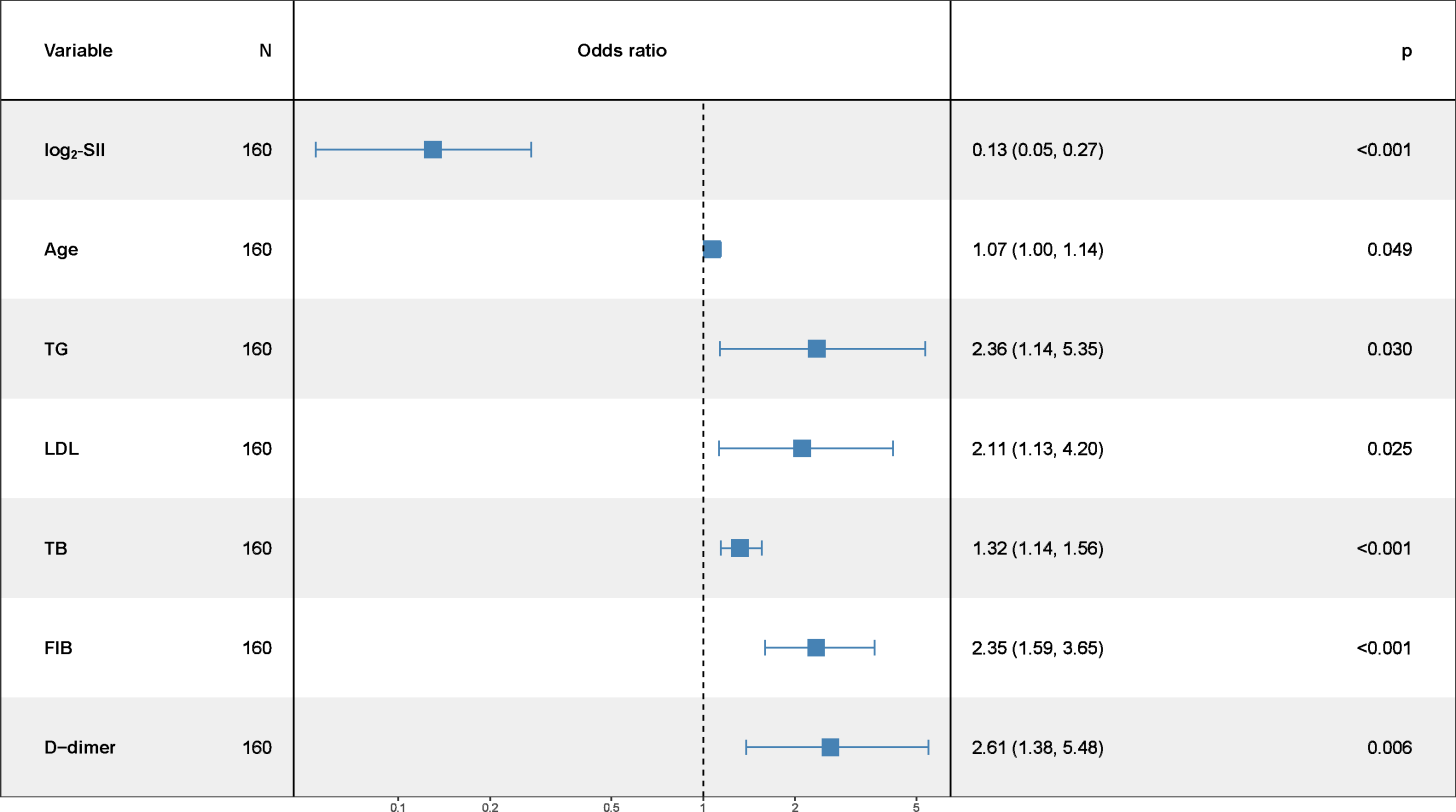
The optimized multifactor logistic regression model of log2-SII. (OR > 1.00 and P < 0.05, indicating that the variable was associated with an increased risk of irH.).

After further screening of variables with a P < 0.05 in univariate regression analysis, multivariate regression analysis was performed using logistic regression. As presented in **Table 3**, the multivariate logistic regression results were summarized as Model 1. To further optimize the predictive model, we performed stepwise backward regression (Model 2). A VIF value more than 10 or a TOL less than 0.1 indicated the presence of multicollinearity. There were no multicollinearity among the variables in the Model 2 (**Supplementary Table 1**). The results (**Figure 2**) showed that log2-SII (OR: 0.13, 95% CI: 0.05-0.27, P < 0.001), age (OR: 1.07, 95% CI: 1.00-1.14, P = 0.049), TG (OR: 2.36, 95% CI: 1.14-5.35, P = 0.030), LDL (OR: 2.11, 95% CI: 1.13-4.20, P = 0.025), TB (OR: 1.32, 95% CI: 1.14-1.56, P < 0.001), FIB (OR: 2.35, 95% CI: 1.59-3.65, P < 0.001), and D-dimer (OR: 2.61, 95% CI: 1.38-5.48, P = 0.006) were significantly associated with the irH risk. The interpretation of the log2-SII OR was as follows: Since log2-SII was log2-transformed SII, a one-unit increase in log2-SII corresponds to a doubling of the raw SII from baseline. The OR of 0.13 meaned that for each doubling of the raw SII, the odds of developing irH were multiplied by 0.13, the odds of irH decreased by 87%.

**Table 3:** Multiple logistic regression analysis.

	Model 1		Model 2	
Variable	OR (95% CI)	P	OR (95% CI)	P
log2-SII	0.11 (0.04, 0.26)	<0.001	0.13 (0.05, 0.27)	<0.001
Age	1.08 (1.01, 1.16)	0.040	1.07 (1.00, 1.14)	0.049
Drinking History				
No	Reference			
Yes	1.34 (0.43, 4.20)	0.610		
TC	0.29 (0.04, 1.61)	0.175		
TG	2.62 (1.03, 7.50)	0.058	2.36 (1.14, 5.35)	0.030
LDL	8.16 (1.34, 66.79)	0.033	2.11 (1.13, 4.20)	0.025
FBG	1.39 (0.96, 2.16)	0.115		
TB	1.34 (1.15, 1.59)	<0.001	1.32 (1.14, 1.56)	<0.001
PT	1.30 (0.76, 2.28)	0.350		
FIB	2.02 (1.34, 3.22)	0.001	2.35 (1.59, 3.65)	<0.001
D−dimer	2.64 (1.39, 5.52)	0.005	2.61 (1.38, 5.48)	0.006

Variables with P < 0.05 in univariate logistic regression were entered into multivariate logistic regression to obtain Model 1. Backward stepwise regression was then used for further optimization to obtain Model 2. SII, systemic immune-inflammation index; TC, total cholesterol; TG, triglycerides; LDL, low density lipoprotein; FBG, fasting blood glucose; TB, total bilirubin; PT, prothrombin time; FIB, fibrinogen.

### 3.4 Nomogram development and evaluation

**Figure 3:**
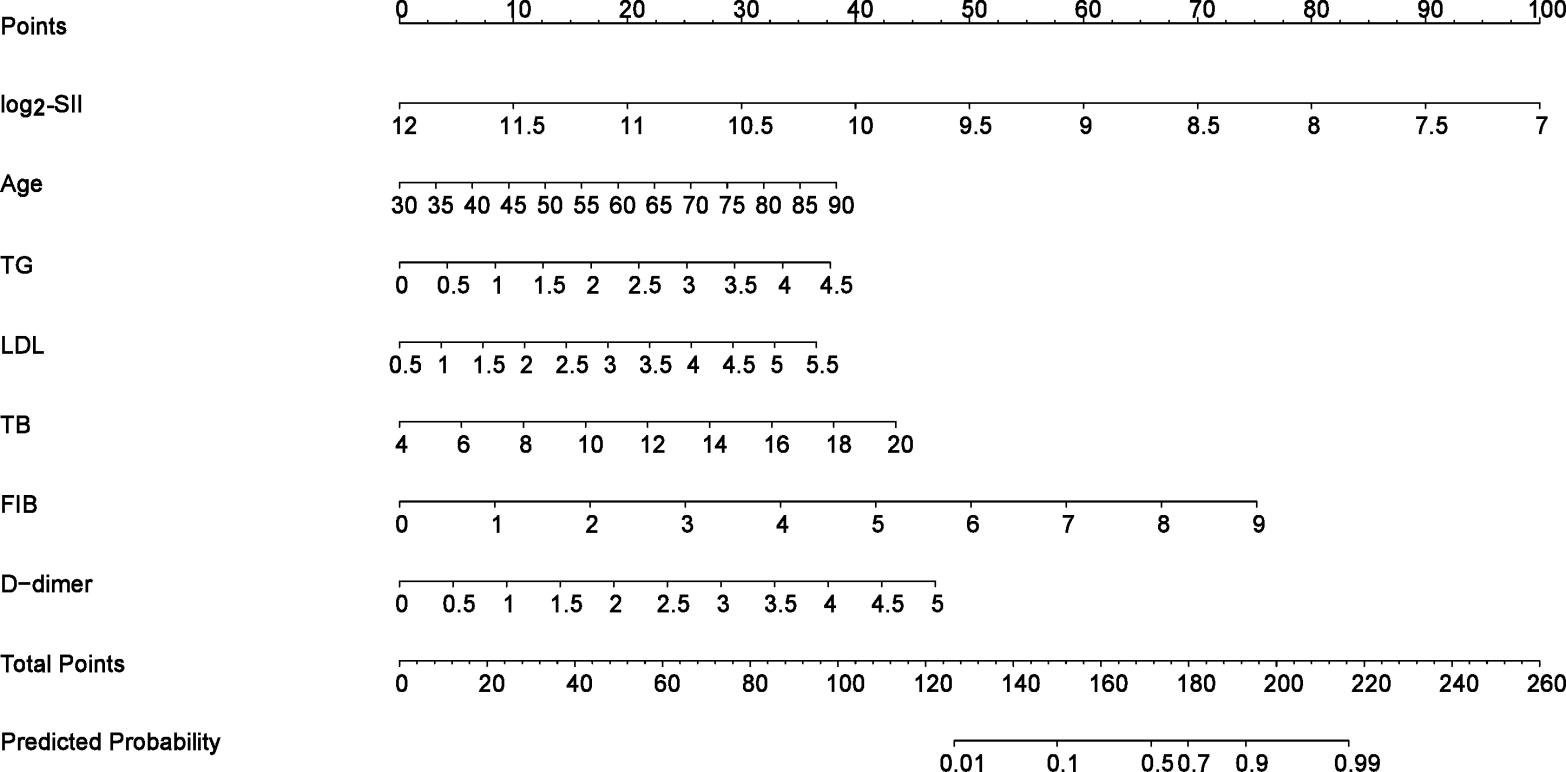
The nomogram for predicting irH. (Points were assigned to each variable based on the patient’s specific indicators, and the sum yielded a total score corresponding to a predicted probability. A higher total score indicated a greater risk of irH).

A nomogram was developed using the results of Model 2 (**Figure 3**). The results indicated that low log2-SII, advanced age, elevated TG, elevated LDL, elevated TB, elevated FIB, and elevated D-dimer were predictors for irH during immunotherapy in patients with NSCLC. When using this nomogram to predict irH risk, each risk factor corresponds to a point on the scale. By summing the points assigned to each risk factor, a total score can be obtained. An increased total score was associated with an elevated risk of irH development during immunotherapy in NSCLC patients.

**Figure 4:**
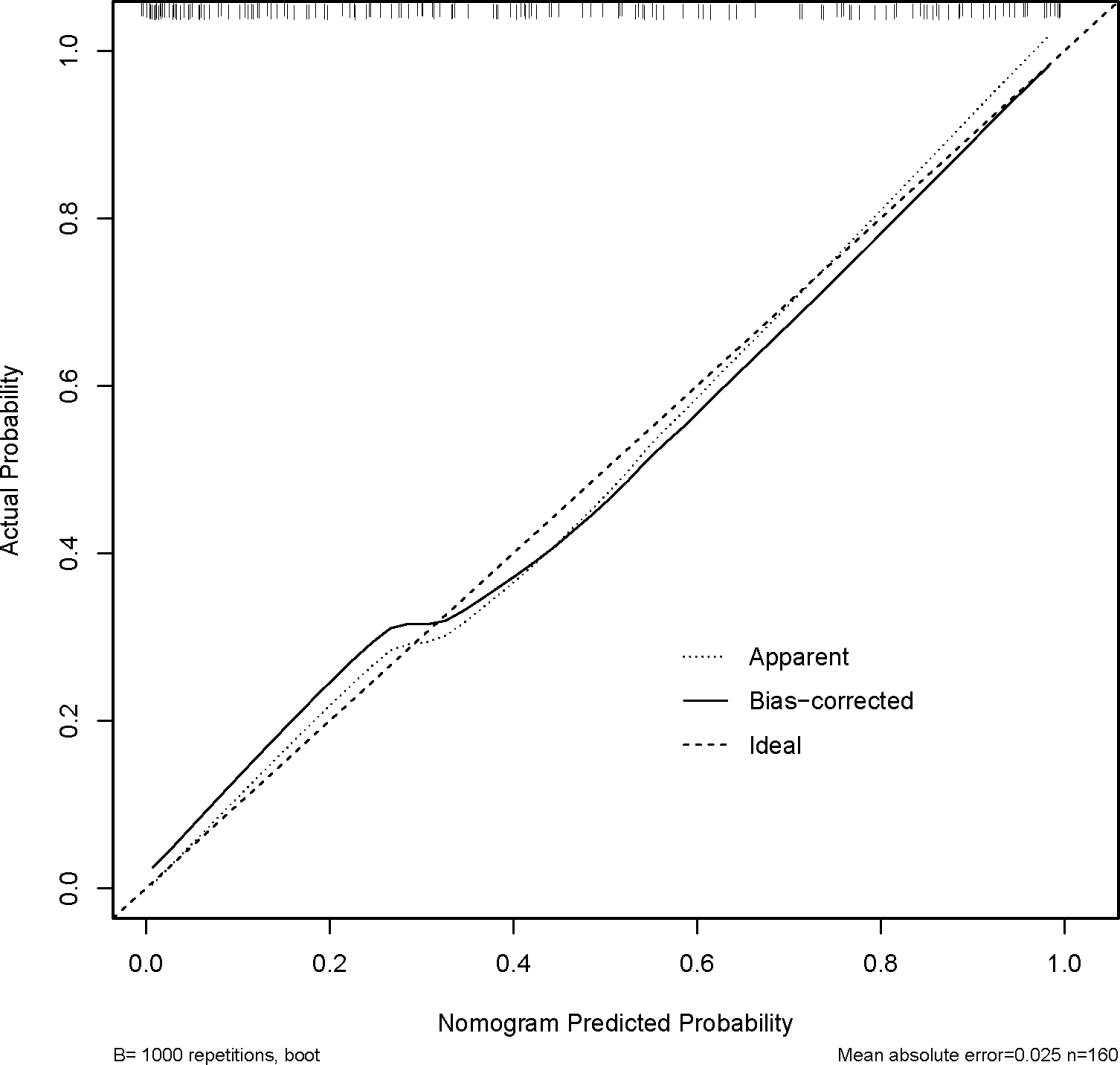
The calibration curve of the nomogram for predicting irH. (The calibration curve was generated using 1,000 bootstrap repetitions. In the plot, the diagonal dashed line represents ideal predictions, while the solid line indicates the actual performance of the model. Greater proximity between the two lines reflects better predictive accuracy).

**Figure 5:**
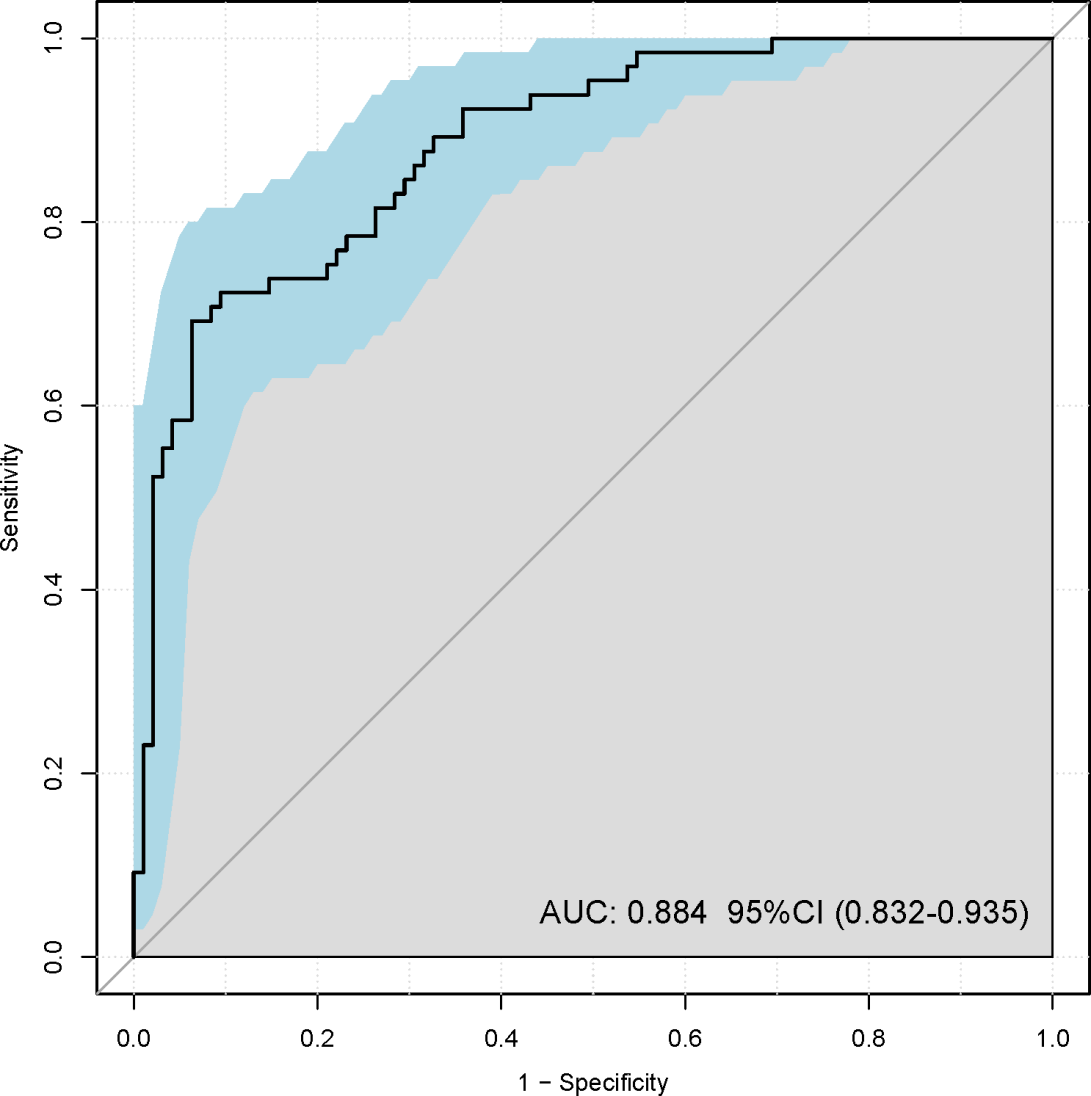
ROC curve of the nomogram for predicting irH. (AUC > 0.7 indicating that the model demonstrates relatively good discriminatory ability).

**Figure 6:**
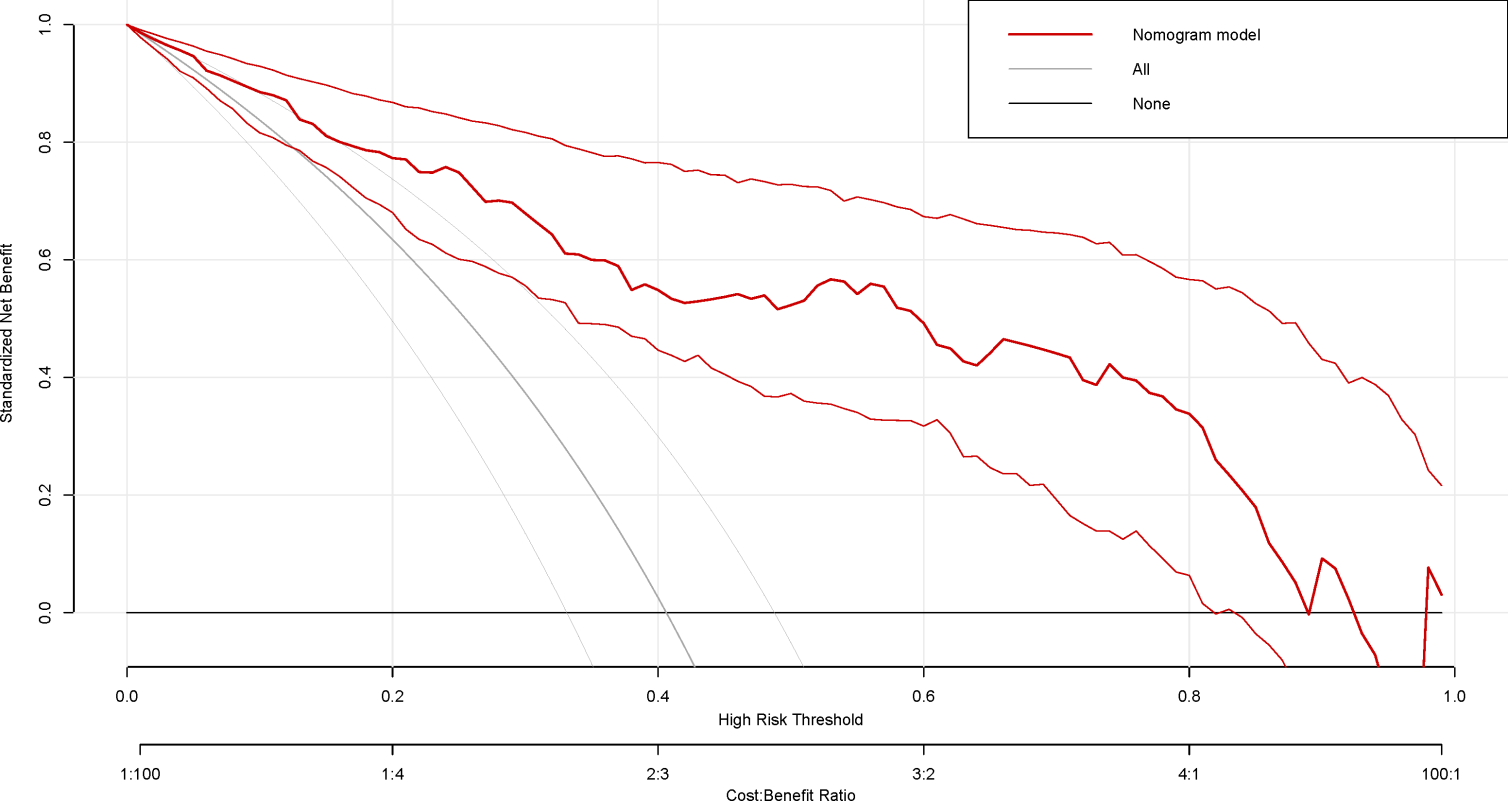
The decision curve of the nomogram for predicting irH. (Across threshold probabilities ranging from 5% to 90%, the nomogram provided greater net benefit than either the treat−all or treat−none strategies, demonstrating its clinical utility in routine practice).

To assess the clinical applicability of this predictive model, a calibration curve (1,000 bootstrap resamples) was employed for validation. The diagonal dashed line denotes the ideal reference, corresponding to perfect prediction, whereas the solid line reflects the actual performance of the model. The closer these two lines are, the better the predictive accuracy of the model. The results are shown in **Figure 4**. The mean absolute error between the bootstrap-corrected curve and the apparent curve was 0.025, indicating a high degree of concordance between the bias-corrected curve and the apparent curve of the model. The nomogram achieved an AUC of 0.884 (95% CI: 0.832-0.935) (**Figure 5**), demonstrating potential discriminative ability. DCA confirmed that the nomogram offered a superior net clinical benefit over both the “treat-all” and “treat-none” strategies across the entire threshold probability range (5%–90%), thereby demonstrating its clinical utility (**Figure 6**).

### 3.5 Subgroup analysis

In order to further explore the expression levels of SII in different pathological types, subgroup analyses were performed (**Table 4**). Subgroup analyses showed that in both LUSC and LUAD, the log2-SII values were reduced in the irH group compared with the non-irH group (all P<0.05). Additionally, the results of a significant negative correlation were obtained through logistic regression analysis between SII level and the risk of irH (LUSC: OR = 0.32, 95% CI: 0.14-0.72, P = 0.006; LUAD: OR = 0.32, 95% CI: 0.17-0.62, P = 0.001).

**Table 4:** Subgroup analysis.

Pathological type	Non-irH group	irH group	P	OR (95% CI)	P
LUSC	9.56 ± 0.76	8.95 ± 0.70	0.002	0.32 (0.14, 0.72)	0.006
LUAD	9.55 ± 0.83	8.94 ± 0.64	<0.001	0.32 (0.17, 0.62)	0.001

irH, immune-related hypothyroidism; LUSC, lung squamous cell carcinoma; LUAD, lung adenocarcinoma.

In addition, we examined SII and log2−SII levels among patients with different hypothyroidism statuses (**Table 5**). Among irH patients, 46 (70.77%) had primary hypothyroidism and 19 (29.23%) had subclinical hypothyroidism. Compared with the subclinical hypothyroidism group, the primary hypothyroidism group had lower SII (434.72 ± 248.81 vs 588.68 ± 219.74, P = 0.026) and log2-SII levels (8.56 ± 0.76 vs 9.11 ± 0.54, P = 0.009).

**Table 5:** Comparison of SII and log2−SII between subclinical and primary hypothyroidism.

Variable	Subclinical hypothyroidism group (n=46)	Primary hypothyroidism group (n=19)	P
SII	588.68 ± 219.74	434.72 ± 248.81	0.026
log2-SII	9.11 ± 0.54	8.56 ± 0.76	0.009

## 4 Discussion

Our research results indicated that among NSCLC patients who received ICI therapy, lower baseline SII levels were associated with an increased risk of irH occurrence. Therefore, these findings provide a potential biomarker for identifying patients with a high risk of irH in clinical practice. SII is a comprehensive index that provides a more scientific evaluation of the overall inflammatory and immune condition of the body than individual indicators (20). SII actually reflects the complex interactive effects of neutrophils, platelets, and lymphocytes.

To further elucidate the drivers of low SII in patients with irH, we compared the three components of SII. The irH group demonstrated higher baseline lymphocyte counts, together with lower platelet and neutrophil counts, compared with the non-irH group. Lymphocytes are core effector cells of adaptive immunity, particularly CD8^+^ cytotoxic T cells and CD4^+^ helper T cells (21). Higher baseline lymphocyte counts suggest that patients possess a relatively intact or enhanced adaptive immune reserve. Following immune checkpoint inhibitor (ICI) therapy, the suppressed T cells become rapidly activated, clonally expand, and infiltrate the tumor microenvironment, thereby exerting antitumor effects (22, 23). However, excessively activated T cells may also recognize autoantigens in thyroid tissue (e.g., thyroglobulin, thyroid peroxidase), leading to damage to thyroid follicular epithelial cells and consequently inducing irH (24). Neutrophils are primary effector cells of acute inflammation. In the tumor microenvironment, they can be polarized into the N2 phenotype by factors such as TGF-β, subsequently secreting arginase−1 and reactive oxygen species that suppress T cell function, thereby establishing an immunosuppressive state (25, 26). Decreased neutrophil count implies a relatively low inflammatory state, characterized by the absence of an overactive innate immune response, thereby avoiding neutrophil−mediated suppression of T cell function (27, 28). Concurrently, it reduces the negative regulation of effector T cells, allowing more complete activation of ICI−induced T cells. The reduced immunosuppressive signals create conditions for ICIs to break immune tolerance and trigger autoimmune reactions (29, 30). Platelets regulate immune responses by releasing mediators such as TGF−β, P−selectin, and CD40L. Platelet−derived TGF−β can suppress the proliferation and function of CD4^+^ and CD8^+^ T cells and promote the differentiation of regulatory T cells, thereby attenuating the immunostimulatory effects of ICIs (31, 32). Furthermore, platelets can directly inhibit T cell activity through the expression of PD−L1 (33). Therefore, a high SII usually indicates a suppressive state of “high inflammation, low immunity”, whereas a low SII shows a relatively advantageous state of “low inflammation, strong immunity”. In this context, following the relief of CTLA-4/PD-1 inhibition by ICIs, T cells undergo substantial expansion and activation. These T cells not only effectively kill tumor cells but also readily attack thyroid tissue containing autoantigens, ultimately manifesting as irH.

Subgroup analysis by pathological type (LUSC and LUAD) revealed that the association between low baseline SII and increased risk of irH was consistent across both subtypes. This consistency is plausible because SII is a systemic inflammatory marker that reflects the host’s global immune−inflammatory status rather than tumor−specific local features (24, 34). Although LUSC and LUAD differ in their tumor microenvironments (e.g., mutation burden, immune cell infiltration patterns) (35), the development of endocrine irAEs such as irH is primarily driven by systemic T−cell activation following ICI therapy, which is not restricted by the histological origin of the tumor (36). Therefore, a systemic marker like SII would be expected to show a similar predictive relationship with irH across different NSCLC pathological types. The antitumor mechanism of ICIs involves eliminating inhibitory effects on T cells due to tumors, thereby reactivating and expanding effector T cells to restore an effective antitumor immune response (37, 38). When the human body is in a low inflammatory condition, the immunostimulatory effects of ICIs may be more rapid and obvious. While this highly activated immune system can kill tumor cells effectively, it may also cause an overactivated state because of disordered immune homeostasis, thus leading to attacks on normal tissues and the occurrence of irAEs (39). As an organ that is often affected, the thyroid usually presents as irH (40). Studies have verified a positive association between the occurrence of irAEs (including irTD) and the antitumor effect of ICIs, which suggests that both phenomena share the core immunological mechanism of T-cell activation (41, 42). Hence, existing research supports these findings. A study on metastatic urothelial carcinoma has indicated that low baseline SII corresponded to a higher likelihood of overall irAEs after ICI therapy (P = 0.01) (43). Similarly, Yalcin et al. confirmed that among patients with metastatic tumors who received ICIs, a high SII was associated with poorer prognosis, and these patients had fewer irAEs (44). These findings jointly support the idea that patients with low baseline SII have a higher risk of developing irAEs, which reflects an intrinsic connection between efficacy and toxicity that is mediated by immune activation mechanisms.

Clinical studies have also reported relations between other inflammatory markers and irAEs. A retrospective analysis in advanced cancer patients showed that individuals who developed hypothyroidism had significantly lower baseline NLR levels than those without hypothyroidism (P = 0.006), indicating that low baseline NLR may be related to the development of irH (45). Another study that investigated factors influencing irTD in advanced gynecological malignancies showed that low baseline platelet-to-lymphocyte ratio (PLR) was an independent protective factor for irTD, which means that lower PLR levels were linked to a higher risk of irTD (46). Collectively, the available evidence from different angles points to a central trend: patients with lower baseline inflammatory levels are more likely to have immune system activation after ICI therapy and accordingly face a higher risk of developing irAEs.

In addition, the study also identified age, TG, LDL, TB, FIB, and D-dimer as risk factors for irH during immunotherapy in NSCLC. Consistently, Ata et al. also reported that elderly patients were more likely to suffer from hypothyroidism (47), which may be related to reduced thyroid functional reserve and disturbed immune homeostasis in older people. Hyperlipidemia can activate oxidative stress responses (48), while elevated baseline FIB and D-dimer levels often show a hypercoagulable state (49), which makes patients more prone to developing microcirculatory thrombosis and subsequent hypoxic damage to thyroid tissue. These conditions may also strengthen oxidative stress, which can damage the normal function of thyroid follicular epithelial cells, promote the production of autoantigens (50, 51), and reduce the immunosuppressive function of regulatory T cells (52). These processes together enhance immunogenicity and reduce immune tolerance to self-tissues, thereby increasing the susceptibility to immune-mediated thyroid injury.

However, this study has several limitations. First, as a single-center study in which all the patients were recruited from the same institution, external validation could not be performed. There may be significant homogeneity in terms of regional distribution, treatment plans, etc. among our study population, thus restricting the generalizability of our findings to wider populations. Second, the limited sample size (n = 160) may influence the stability of the model and the reproducibility of our results. Furthermore, the limited sample size restricted subgroup analyses and reduced statistical power. Third, the retrospective design of this study means we had to rely on the completeness and accuracy of historical medical records, which may have introduced potential selection or information bias. Fourth, although we tried to control known confounders in our analysis, the possibility of residual confounding due to unmeasured factors remains. Fifth, blood samples were collected only at baseline before immunotherapy initiation; post-irH levels of inflammatory markers, lipids and coagulation parameters were not measured. Therefore, we could not assess whether hypothyroidism itself altered these parameters. These limitations provide some directions for future work. First, the association between SII and irH, and the predictive performance of the model will need confirmation from large-scale, multi-center prospective studies. Second, to assess generalizability, our findings will require external validation in different patient populations and clinical settings. Third, adding other biomarkers into the prediction model (such as inflammatory cytokines and genetic markers) will strengthen the predictive completeness and clinical applicability of the model.

## 5 Conclusion

In summary, this study presented a systematic exploration into the relationship between baseline SII levels and irH in the NSCLC population for the first time. The findings indicated that baseline SII was a potentially useful predictive indicator for irH. Patients with low baseline SII exhibited a higher risk of irH. According to this, clinicians should enhance thyroid function monitoring in patients with low baseline SII following the initiation of ICI therapy, allowing for earlier detection and intervention of irH.

## Data Availability

The original contributions presented in the study are included in the article/**Supplementary Material**. Further inquiries can be directed to the corresponding authors.
