# Supplementary material for: Associations between systemic immune-inflammation index and immune-related hypothyroidism in non-small cell lung cancer patients receiving immune checkpoint inhibitors: a retrospective study
Source: Front Oncol. 2026 Jun 2;16:1844002. doi: 10.3389/fonc.2026.1844002 (PMC13268993; doi:10.3389/fonc.2026.1844002)
Supplement: Supplementary file 1 [file Table1.docx]

Table S1 Multicollinearity detection inoptimized multiple logistic regression model

| Variable | TOL | VIF |
| --- | --- | --- |
| log2-SII | 0.909 | 1.100 |
| Age | 0.884 | 1.131 |
| TG | 0.923 | 1.083 |
| LDL | 0.926 | 1.080 |
| TB | 0.890 | 1.123 |
| FIB | 0.834 | 1.200 |
| D-dimer | 0.911 | 1.098 |

TOL, Tolerance; VIF, Variance inflation factor

A VIF value more than 10 or a TOL less than 0.1 indicate multicollinearity.
